# Electrospraying and Nano‐Enabled Edible Films for Food Preservation: Controlled Release, Antimicrobial Function, and Future Horizons

**DOI:** 10.1002/fsn3.72081

**Published:** 2026-07-11

**Authors:** Messenbet Geremew Kassa, Eshetie Gelagay Erku, Desye Alemu Teferi

**Affiliations:** ^1^ Department of Food Science and Post‐Harvest Technology Injibara University Injibara Ethiopia; ^2^ College of Agriculture, Food, Climate Science Injibara University Injibara Ethiopia

**Keywords:** antimicrobial packaging, controlled release, electrospraying, food preservation, nano‐enabled edible films

## Abstract

Food preservation stands as the primary obstacle that needs resolution to achieve safe and quality food maintenance. The food preservation field is developing new methods through the combination of electrospraying technology and nano‐enabled edible films which create advanced food preservation methods. The electrospraying process serves as a flexible method that enables manufacturers to create micro and nano‐structured coatings which function as delivery systems for bioactive substances with controlled release capabilities. The mechanical strength and barrier resistance, antibacterial effectiveness, and overall performance of edible films get enhanced through the integration of nano materials and nano carriers in their production. This study analyzes the fundamental electrospraying principles and the development of nano‐enabled edible films and their implementation in food preservation through controlled release and antimicrobial functions. The review examines the mechanisms of action together with biopolymer and nanomaterial components used in electrospraying and nano‐enabled edible films during their implementation in different food applications. The safety and regulatory aspects of electrospraying and nano‐enabled edible films are also discussed. The combination of electrospraying and nano‐enabled edible films creates an innovative method that enables sustainable food preservation together with new functional packaging solutions.

## Graphical abstract

This graphical abstract illustrates the integration of electrospraying technology and nano‐enabled edible films as an innovative strategy for food preservation. Electrospraying utilizes a high‐voltage electric field to generate fine droplets from polymer‐bioactive solutions, resulting in the formation of nano‐sized particles and uniform edible film coatings. The incorporation of nanocarriers and bioactive compounds within the film matrix enhances functionality by enabling the controlled release of active ingredients such as antimicrobials, antioxidants, and natural preservatives. These nano‐enabled films provide antimicrobial protection by inhibiting microbial growth and disrupting pathogen activity, thereby extending food shelf life and improving food safety. In addition, the films serve as effective barrier systems against moisture, oxygen, and lipid oxidation, helping to maintain the physicochemical and sensory quality of food products. Applications span a wide range of food systems, including fruits, vegetables, meat, dairy, bakery, and ready‐to‐eat products. Looking ahead, advances in sustainable biopolymers, smart responsive packaging, scalable electrospraying technologies, and multifunctional nano‐delivery systems are expected to drive the next generation of edible packaging solutions, contributing to reduced food waste, enhanced product quality, and improved sustainability in the food industry.

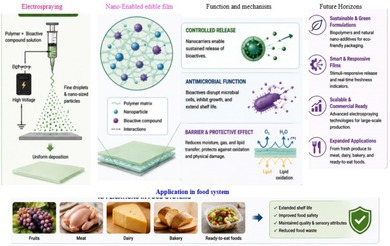

## 1 Introduction

The global food sector faces major challenges due to unsafe food, declining food quality, and poor postharvest preservation, threatening food security and economic stability (Osei‐Kwarteng et al. 2024). Food products lose shelf life and sensory quality mainly due to microbial spoilage, oxidative degradation, and moisture and gas exchange (Mercanti et al. 2024). The research for new food preservation technologies has increased due to concerns about chemical residues, environmental pollution, and the demand for clean‐label and sustainable products (Lisboa et al. 2024). Traditional food preservation techniques, such as refrigeration, chemical preservatives, and conventional plastic packaging, have been somewhat successful (Lisboa et al. 2024).

Edible films and coatings have emerged as sustainable alternatives to traditional packaging due to their biodegradability, edibility, and multifunctional properties (Nunes et al. 2023). Edible films made from natural biopolymers, including proteins, lipids, and polysaccharides, act as semi‐permeable barriers that reduce moisture, oxygen, and solute transfer, thereby preserving food quality and extending shelf life (Tavassoli‐Kafrani et al. 2022). Edible films can act as active carriers for functional compounds, such as antimicrobials, antioxidants, and nutraceuticals, in addition to their passive barrier function (Moeini et al. 2022). Encapsulation of bioactive compounds like essential oils, plant extracts, and microbial metabolites protects them from degradation caused by light, oxygen, temperature, and moisture (Bae et al. 2022). This technology enhances the stability, biological integrity, controlled release, and long‐term viability of active ingredients during storage and application. In agricultural and food systems, encapsulation enhances formulation stability, reduces volatilization losses, and improves the effectiveness and usability of bioactive agents (Meftah Kadmiri et al. 2021). Various biopolymers are used as wall materials for encapsulation, including chitosan, gums, maltodextrin, pectin, starch, whey protein, alginate, cellulose derivatives, zein, pullulan, galactomannan, and sodium caseinate (Ali et al. 2021; Castejón et al. 2021). The direct application of bioactive agents is limited by poor stability, rapid release, reduced effectiveness, and undesirable sensory effects (Wang, Xue, et al. 2024).

Recent advances in nanotechnology have led to edible films with improved performance through nano‐enabled systems that enhance mechanical strength, barrier properties, and bio‐functional activity (Mahato et al. 2024). Edible films incorporating nanomaterials and nanostructured carriers improve the distribution, stability, bioavailability, and controlled release of active compounds (Pascuta and Vodnar 2022). Nano‐enabled edible films show improved antimicrobial activity against foodborne pathogens and spoilage microorganisms, along with enhanced antioxidant and protective properties against environmental factors (Wang, Guo, et al. 2025). These properties make them powerful tools for active food packaging and preservation (Li et al. 2025).

In this context, electrospinning is an effective method for encapsulating heat‐sensitive reactants (Jayaprakash et al. 2023). The system allows full control over operating parameters, enabling the production of nano‐carriers tailored for specific controlled‐release applications (Wang and Zhou 2025). Combining edible films with electrospraying enables surface functionalization while preserving the material's overall properties (Dhiman et al. 2022). This is a significant advantage over other conventional approaches to material fabrication (Lisboa et al. 2024).

The synergistic combination of electrospraying technology with nano‐enhanced food packaging films serves as a revolutionary idea in food preservation (Younis et al. 2025). This technology creates multifunctional systems that provide barrier, antimicrobial, and antioxidant protection while reducing the need for artificial chemical additives (Hao et al. 2025). The electrosprayed nano‐carriers function as smart systems that utilize environmental factors such as moisture, pH, and temperature changes to detect alterations in food products (Haider et al. 2026). With such potential, the technology is of enormous importance in preserving perishable foods like fresh produce, meats, poultry, and dairy products (Peng et al. 2023).

The large‐scale use of electrospraying and nano‐enabled edible films is limited by scalability challenges, regulatory approval requirements, and concerns about nanomaterial migration and safety (Mahajan et al. 2025). This review examines the fundamentals of electrospraying and evaluates nano‐enabled edible films for food preservation. It also discusses their challenges, safety issues, and future research directions in sustainable food systems.

## 2 Methodology

**FIGURE 1:**
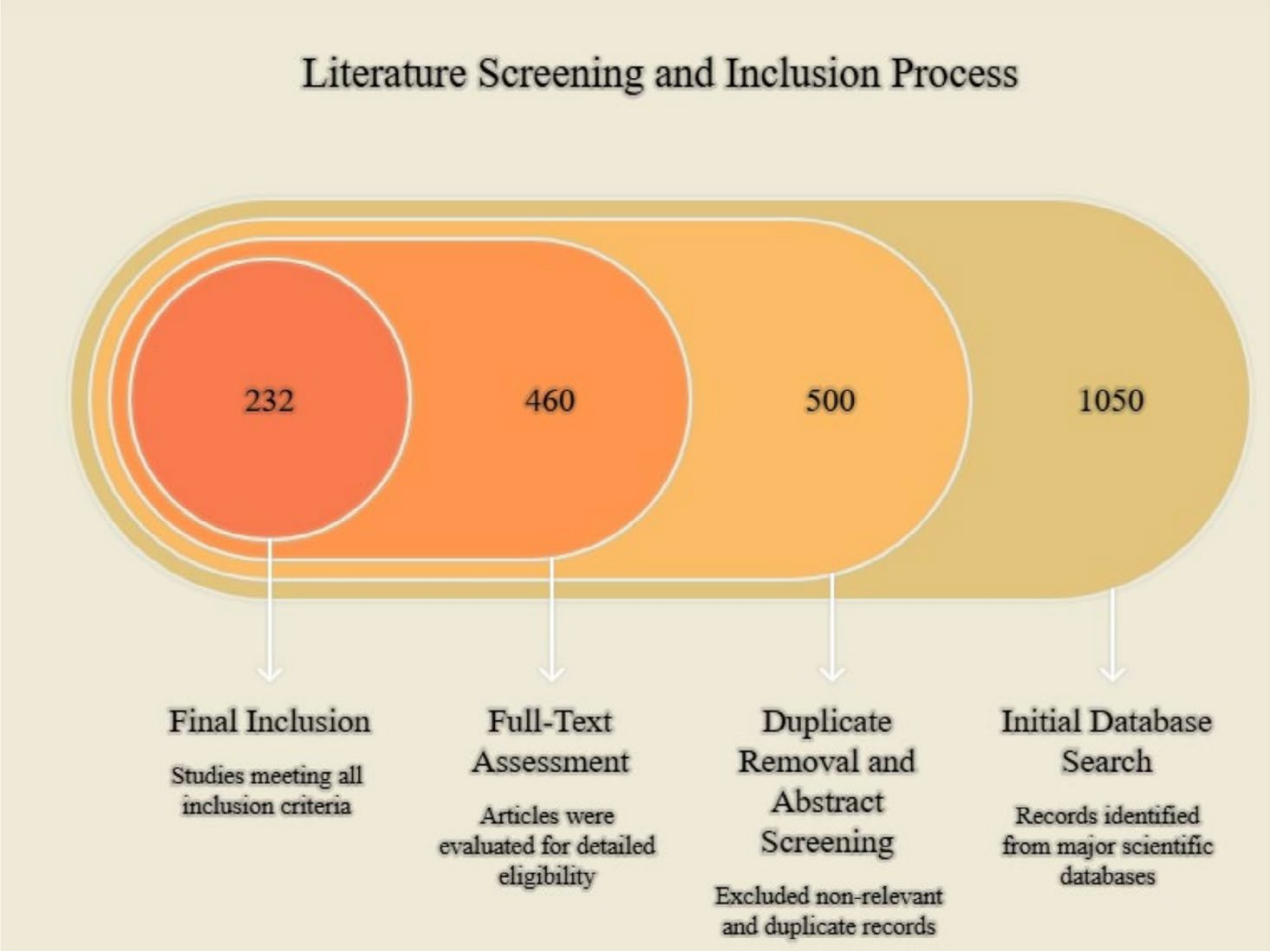
Electrospraying and nano‐enabled edible films for food preservation literature review process.

This review adopted a systematic approach to collect, evaluate, and synthesize scientific evidence on the application of electrospraying and nano‐enabled edible films for food preservation, with a particular focus on controlled release mechanisms, antimicrobial functionality, and future research prospects (Figure 1). Relevant literature was retrieved from major scientific databases, including Scopus, Web of Science, PubMed, Science Direct, and Google benefits “electrospraying” AND “food preservation”, “nano‐enabled edible films” AND “controlled release”, and “antimicrobial edible films” OR “active food packaging.” Scholar, covering publications up to 2026. A comprehensive search strategy was applied using Boolean operators (“AND” and “OR”) to combine keywords such as “electrospraying” AND “food preservation,” “nano‐enabled edible films” AND “controlled release,” “antimicrobial edible films” OR “active food packaging,” and “encapsulation” AND “bioactive compounds” AND “food applications.

A total of 1050 articles were initially identified through database searching. After removing duplicates and conducting preliminary screening, 500 studies were retained based on title and abstract relevance. Subsequently, 460 articles underwent full‐text assessment according to predefined eligibility criteria, resulting in the final inclusion of 232 studies for comprehensive analysis (Table 1).

**TABLE 1:** Summary of literature screening and inclusion process.

Stages	Description	Number of articles
Initial database search	Records identified from Scopus, Web of Science, Science Direct, PubMed, and Google Scholar	1050
Duplicate removal and abstract screening	Excluded non‐ relevant and duplicate records	500
Full‐ text assessment	Articles were evaluated for detailed eligibility	460
Final inclusion	Studies meeting all inclusion criteria	232

The study selection process was guided by principles adapted from Bloom's Taxonomy, enabling structured and comprehensive coverage of the literature. Foundational studies describing the principles, mechanisms, and fabrication techniques of electrospraying and nano‐enabled edible films were included to support knowledge acquisition and understanding. Studies evaluating their applications in food preservation, controlled release systems, antimicrobial functionality, and shelf‐life extension were selected to facilitate analysis and application. In addition, recent research addressing performance evaluation, safety considerations, scalability challenges, regulatory aspects, and future innovations was incorporated to support higher‐order evaluation and synthesis of current knowledge in the field.

Only peer‐reviewed articles, review papers, and book chapters published in English were included, whereas duplicate records, non‐scientific reports, and irrelevant studies were excluded. Articles were screened based on titles, abstracts, and full texts to ensure relevance and quality.

Key data extracted from the selected studies included film composition, type of biopolymer and nanomaterial used, electrospraying parameters, encapsulated bioactive compounds, particle size and morphology, encapsulation efficiency, controlled release behavior, antimicrobial and antioxidant performance, barrier and mechanical properties, food application outcomes, shelf‐life extension effects, safety considerations, and reported limitations or future research directions. These data were systematically analyzed to evaluate the effectiveness and potential of electrospraying and nano‐enabled edible films in food preservation.

## 3 Fundamentals of Electrospraying Technology

### 3.1 Principle of Electrospraying

Electrospraying is an electrohydrodynamic atomization technique that produces micro‐ to nanosized droplets by applying a high electric field to liquid solutions (Dhiman et al. 2022). The method works by balancing electrostatic and surface tension forces in a polymer solution flowing through a capillary nozzle (Granda et al. 2025). Electrospraying is governed by the balance between electrostatic forces and surface tension at the liquid–air interface (Dhiman et al. 2022). Under a high electric field, surface charges of the same polarity accumulate on the liquid emerging from the nozzle, generating Coulombic repulsion that opposes surface tension (Garcia et al. 2023). When electrostatic stress exceeds capillary forces, the liquid deforms into a Taylor cone and emits a charged jet (Garcia et al. 2023). Coulombic repulsion continuously destabilizes the jet, causing stretching, thinning, and charge‐driven elongation in the cone–jet regime (Li et al. 2025). As the jet travels, electrohydrodynamic instabilities develop, leading to primary breakup into charged micro‐ or nanodroplets (Wang, Kong, et al. 2022). If the charge exceeds the Rayleigh limit, further Coulombic fission occurs, producing smaller daughter droplets. Finally, solvent evaporation solidifies the particles, enabling controlled formation of micro‐ to nanoscale structures. Applying high voltage between the nozzle and grounded collector generates electrostatic charges on the liquid surface. This charge accumulation results in the liquid meniscus transforming into a conical shape known as the Taylor cone (Moreira et al. 2024). The balance between electrostatic and surface forces breaks the surface tension, forming a charged liquid jet from the tip of the Taylor cone (Ronizi et al. 2022). The liquid jet then breaks into dispersed droplets because of Coulombic repulsion (Wang, Xue, et al. 2024). The solvent rapidly evaporates during the process as particles reach the collector, leading to the formation of micro‐ to nanosized particles depending on solution properties (Bahndral et al. 2025). The size of the particles depends on various parameters, including solution viscosity, conductivity, surface tension, applied voltage, solution flow rate, and nozzle‐collector distance (Panditkar et al. 2026).

Electrospraying is attractive because it can operate under room conditions, enabling the processing of heat‐sensitive bioactive materials such as antimicrobials, antioxidants, and nutraceuticals (Jayaprakash et al. 2023). For food applications, electrospraying offers the possibility to precisely deposit functional coatings and nano‐carriers onto food films without affecting their physical integrity (Aruna et al. 2024). The control over the process for the generation and deposition of particles is highly efficient in electrospraying, so that the technique is ideal for the development of advanced nano‐enabled food films.

### 3.2 Process Parameters Affecting Electrospraying

The electrospraying process achieves its operational effectiveness and stable performance through the influence of various solution parameters, processing settings, and environmental conditions, which determine the properties of produced particles (Wang, Guo, et al. 2025). Applied voltage is a key factor, as it governs Taylor cone formation and transitions the system from dripping mode to a stable cone‐jet operation (Yang et al. 2022). Insufficient voltage results in unstable spraying, whereas excessively high voltage may lead to jet instabilities and broad particle size distributions (Wang, Kong, et al. 2022). Therefore, an optimal voltage range is essential for producing uniform micro‐ or nano‐sized droplets (Han et al. 2025). The flow rate of the polymer solution influences particle size and encapsulation efficiency. Lower flow rates produce smaller, more uniform particles due to sufficient time for solvent evaporation. The higher flow rates of the process result in the creation of larger droplets and develop into non‐uniform shapes (Hernández‐Giottonini et al. 2024). The solution properties require equal attention because they include viscosity, surface tension, and electrical conductivity. The low‐viscosity solutions create fine droplets, but the extremely low viscosity causes spray instability (Yang et al. 2022). Jet formation requires low‐viscosity materials, while high‐viscosity solutions hinder droplet formation and instead produce fiber‐like structures (Rezaeinia et al. 2025). Electrical conductivity controls charge accumulation on the jet, thereby influencing droplet breakup and particle size.

The distance between the nozzle and collector affects the time required for solvent evaporation and the time needed for particles to solidify into solid form. Short distances may result in wet particle deposition and particle agglomeration, whereas excessively long distances can reduce deposition efficiency (Laad and Ghule 2022). The study found that different polymer concentrations affected both particle integrity and encapsulation efficiency, which resulted in collapsed particles at low concentrations and well‐defined spherical structures at high concentrations (Wang, Guo, et al. 2025). Solvent evaporation rates and particle morphology are also influenced by environmental factors such as temperature and relative humidity (Miles et al. 2023). The precise control and optimization of these parameters are required to achieve reproducible electrospraying performance in food applications that need uniform nano‐carriers and controlled release functionality.

### 3.3 Advantages of Electrospraying in Food Applications

**FIGURE 2:**
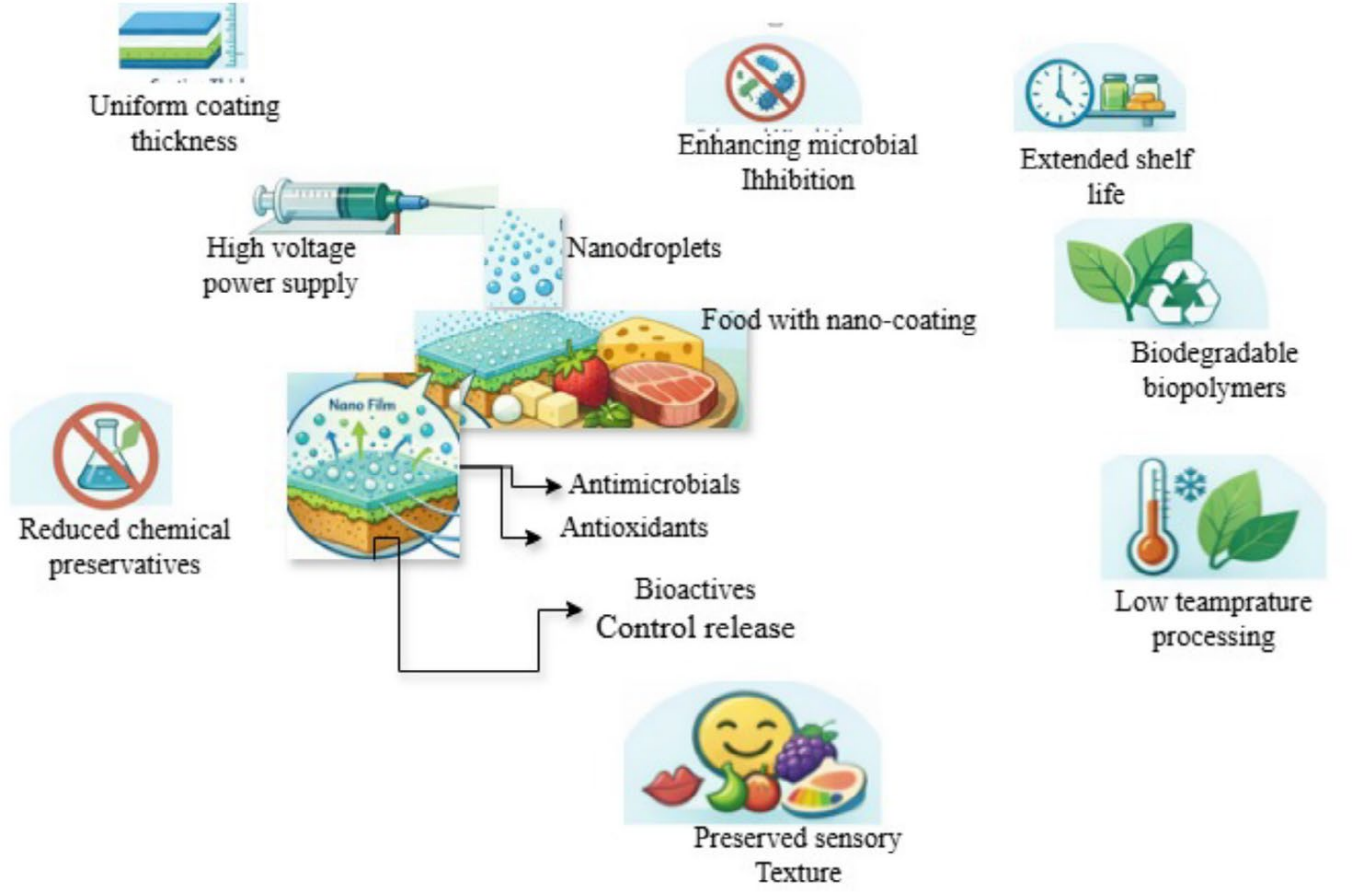
Advantages of electrospraying in food applications.

Electrospraying offers several advantages as a food technology (Figure 2), particularly for the efficient protection and delivery of sensitive bioactive compounds (Wang, Kong, et al. 2022). Electrospraying provides its primary advantage through its ability to function under low temperature requirements. Electrospraying operates at room temperature, unlike conventional encapsulation methods that often require high temperatures and toxic chemicals (Dhiman et al. 2022). The method maintains structural integrity, biological activity, and nutritional value of heat‐sensitive compounds such as vitamins, enzymes, probiotics, polyphenols, and essential oils (Bodbodak et al. 2024).

Electrospraying has been observed to have the key benefits of size and shape control of the particles, thus to meet industrial processes' demands (Ulusoy 2023; Wang, Guo, et al. 2025). The system provides full control over operating conditions, improving encapsulation efficiency, enhancing stability, and enabling tailored release of active compounds in food products (Zabot et al. 2022). This process produces capsules with a high surface area‐to‐volume ratio, which can significantly enhance the solubility and bioavailability of the encapsulated active compounds. This method provides a high surface‐to‐volume ratio, which will be capable of increasing concentration decreases inside the capsules (Klojdová et al. 2023).

Electrospraying is used to spray a variety of food‐grade polymers, such as proteins, polysaccharides, and biodegradable biopolymers (Liu, Zhu, et al. 2025). The technique enables environmental protection through its two main benefits, which include reducing solvent consumption and decreasing material waste (Dhiman et al. 2022). As shown in Figure 2, electrospraying has strong potential as an industrial food processing technology, improving product protection through controlled release, enhanced bioactivity, and maintained safety and quality (Berraquero‐García et al. 2023).

## 4 Nano‐Enabled Edible Films, Materials, and Fabrication

### 4.1 Biopolymer Matrices

Edible films and coatings are thin layers (less than 0.3 mm thickness) made from biopolymers and additives dispersed in aqueous media (Díaz‐Montes and Castro‐Muñoz 2021). Although the terms are sometimes used interchangeably, edible coatings are formed directly on the food surface, while edible films are prepared separately and then applied to the product. In both cases, they form similar protective matrices. Biopolymer matrices serve as the fundamental building blocks for edible films and coatings that function as carriers that transport bioactive substances used in food preservation (Moeini et al. 2022). Biopolymers derived from natural sources such as proteins, polysaccharides, and lipids are biodegradable, non‐toxic, and generally recognized as safe (GRAS), making them suitable for food applications (Benalaya et al. 2024). Protein‐based materials such as gelatin, whey, soy, and zein exhibit excellent film‐forming ability, along with strong mechanical properties and good flexibility (Xing et al. 2026). The materials chitosan, alginate, starch, and cellulose derivatives provide outstanding protection against gas and moisture transmission (Duan et al. 2023). The hydrophobic properties and reduced water vapor transmission which lipid‐based materials bring to products create extended shelf life for products which contain moisture‐sensitive ingredients (Wu et al. 2024).

Biopolymer selection and its blending with other ingredients determine the functional properties to a large extent in the edible films that are obtained (Abdullah et al. 2022). Protein polysaccharide pairs, which constitute multi‐component systems, exhibit synergistic effects that result in improved mechanical strength, increased flexibility, and enhanced controlled release of their embedded bioactive compounds (Meng et al. 2024). Such systems enable the addition of antimicrobial agents, antioxidants, vitamins, and minerals to the film, which increases its functional capabilities and nutraceutical content (Periyasamy et al. 2025). Biopolymer matrices can be processed through various fabrication methods, which include casting, electrospinning, and electrospraying to enable complete control over film thickness, material structure, and encapsulation efficiency (Flórez et al. 2023).

The biopolymer matrices, in general, act as the structural foundations of nano‐enabled foods (Basumatary et al. 2022). In addition, these nanocomposites significantly affect the barrier levels, strength, and the tweaked dispersion of compounds that carry a function (Bahmid and Siddiqui 2024).

### 4.2 Role of Nanomaterials

For the preservation of food, edible films and coatings render functional properties to act as a mechanical barrier, protecting food from the external environment, gas transport, and mass transfer (Bizymis and Tzia 2022). Nanomaterials have a definition that requires them to have one dimension that measures between 1 and 100 nm because this size range results in materials that have increased surface area relative to their volume and display distinct physical and chemical behavior and show greater chemical reactivity than their bulk forms (Mekuye and Abera 2023). In food applications, the use of nanomaterials within biopolymer matrices results in stronger mechanical properties and enhanced barrier performance, better thermal resistance, and improved bioactive delivery efficiency (Zhang, Xue, et al. 2025; Zhang, Qin, et al. 2025; Zhang, Zhang, et al. 2025; Zhang, Liu, et al. 2025). Silver nanoparticles and zinc oxide nanoparticles demonstrate effective antimicrobial properties against various foodborne pathogens, resulting in improved food safety and extended product shelf life (Azam et al. 2022).

Nanomaterials function as dual‐purpose materials because they provide antimicrobial protection and act as effective delivery systems for controlled release of bioactive compounds, which include antioxidants, essential oils, and vitamins (Nair et al. 2022). The nanoscale encapsulation technique protects these delicate compounds from degradation, which occurs through light, heat, and oxygen exposure, while it enables continuous and environmental condition‐based release through pH, moisture, and temperature changes (Tao et al. 2023). Food packaging applications use nanoclays and nanocellulose as materials that strengthen polymer networks, which result in better oxygen and moisture barrier performance and decreased permeability (Jali et al. 2023).

The development of intelligent or responsive packaging systems, which can monitor food quality, detect spoilage, and respond to environmental changes, depends on the special optical, electronic, and catalytic properties of specific nanomaterials (Zhang, Xue, et al. 2025, Zhang, Qin, et al. 2025, Zhang, Zhang, et al. 2025, Zhang, Liu, et al. 2025). Nanomaterials provide vital advantages to edible films because they enhance the films' structural, functional, and bioactive performance, which makes them essential for developing next‐generation food preservation systems that use nanotechnology (Wang, Guo, et al. 2025).

### 4.3 Fabrication Techniques

**FIGURE 3:**
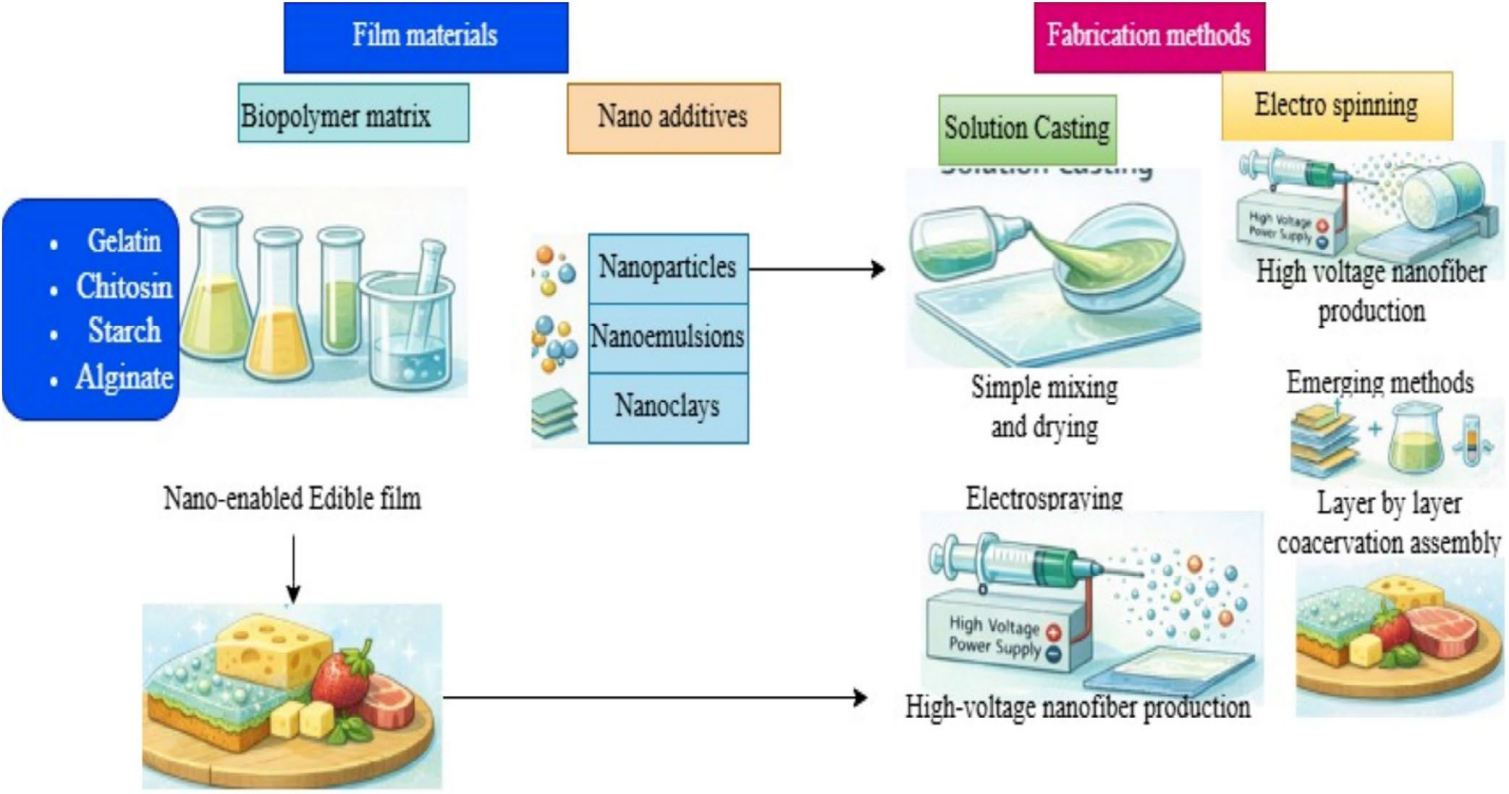
Nano‐enabled films, materials, and fabrication methods.

Nano‐enabled edible films are innovative food packaging materials in which biopolymer matrices are combined with nanomaterials to improve barrier protection, mechanical strength, and bioactive properties (Aditya et al. 2026). As shown in Figure 3, the fabrication of these films involves controlled techniques for incorporating nanoparticles, nanoemulsions, and nanoclays into the polymer matrix to ensure uniform dispersion, structural stability, and functional performance (Peng et al. 2025). The main fabrication methods reported include solution casting, electrospinning, and electrospraying, as well as newer approaches such as layer‐by‐layer assembly and coacervation, each offering specific advantages depending on the application (Si et al. 2024). Layer‐by‐layer assembly and coacervation are considered emerging fabrication techniques for nano‐enabled edible films due to their ability to improve encapsulation efficiency and functional performance (Abad et al. 2021). Layer‐by‐layer assembly involves sequential deposition of oppositely charged biopolymers or nanoparticles to create multilayered structures with improved mechanical, barrier, and controlled release properties (Ganjeh et al. 2024). This method provides precise control over film structure and surface properties, but its multiple deposition steps make it time‐consuming and less suitable for large‐scale production (Richardson et al. 2015). In contrast, coacervation uses phase separation between biopolymers to encapsulate bioactive compounds in polymer‐rich microcapsules, offering high encapsulation efficiency and strong protection for sensitive ingredients such as flavors, probiotics, and essential oils (Napiórkowska and Kurek 2022). Although coacervation is cost‐effective and suitable for certain industrial applications, its stability can be strongly influenced by pH, ionic strength, and processing conditions. Compared with these techniques, electrospraying offers several advantages, including rapid particle formation, mild operating conditions, precise particle size control, and efficient encapsulation of heat‐sensitive bioactive compounds (Jayaprakash et al. 2023).

Recent advances in bioplastics fabrication have improved their mechanical and barrier properties, making them more suitable for food packaging applications. Incorporating nanofillers significantly enhances the overall performance of bioplastics (Kong et al. 2023). Common reinforcement agents include clay, organic, inorganic, and carbon‐based nanomaterials. Organic nanofillers include biopolymers such as chitosan and cellulose, while inorganic nanofillers include metals and metal oxides such as silver, gold, ZnO, and TiO_2_ (Othman and Fadzil 2021). Gelatin‐chitosan (CS) is one of the most widely studied biopolymers for edible films and coatings, especially for fresh fish preservation (Figure 3). It has excellent film‐forming ability, biodegradability, biocompatibility, and strong antimicrobial and antioxidant properties (Eranda et al. 2024). CS is produced from chitin and consists of D‐glucosamine and N‐acetyl‐D‐glucosamine units linked by β‐(1,4) glycosidic bonds (Yang et al. 2021). Its molecular weight, solubility, and degree of deacetylation vary depending on the source and extraction method. Chitosan becomes positively charged and soluble under acidic conditions (pH below 6.5) due to protonation of amino groups. It can be formed into films, gels, and nanoparticles (Shi et al. 2016). The film‐forming ability of chitosan is mainly influenced by its molecular weight and degree of deacetylation, while hydrogen bonding and Van der Waals interactions contribute to the formation of strong films during solvent evaporation (Cazón et al. 2017). Sustainability is an important factor in filler selection, and bio‐based fillers are preferred because they are biodegradable, environmentally friendly, less toxic, and help reduce carbon emissions (Melro et al. 2020). In addition, the compatibility between biopolymers and fillers strongly influences the performance and sustainability of reinforced bioplastics (Jamróz et al. 2019). Solution casting is the simplest and most frequently used form of technique (Zhao et al. 2025). The method involves dissolving a biopolymer such as gelatin, chitosan, starch, or alginate in a suitable solvent, then dispersing nanomaterials or bioactive compounds into the resulting solution (Nishimoto‐Sauceda et al. 2022). The mixture is applied to a flat surface, which can be either a petri dish or a glass plate, and then dried at specific temperature and humidity levels until it becomes a thin film (Kramar et al. 2023). This method allows precise control of film thickness, composition, and nanoparticle distribution, resulting in films with consistent barrier performance and mechanical strength (Shapi'i et al. 2022). Silver and zinc oxide nanoparticles and nanoclays function as antimicrobial agents because they improve the oxygen and moisture barrier properties together with the structural strength of the resulting films (Mafe et al. 2024). Industrial production faces difficulties when expanding solution casting because of two main challenges, which include extended drying periods and the need to manage solvents (Weng et al. 2025).

Electrospinning can simply be described as the use of very high‐voltage electrostatic forces that pull polymer solutions into continuous nanofibers (Al Saif and Cselkó 2025). The process produces fibrous mats of ultrafine fibers with high porosity and a large surface area‐to‐volume ratio, enabling controlled release of bioactive compounds (Tiong et al. 2026). Electrospinning allows the incorporation of heat‐sensitive additives such as essential oils, vitamins, and probiotics without losing their functional properties due to mild processing conditions (Sumnu and Yazicioglu 2025). The resulting nanofibrous films exhibit enhanced mechanical properties, improved oxygen and moisture barriers, and better bioactive delivery efficiency compared to conventional cast films. The films show improved mechanical strength and better barrier protection against oxygen and moisture, and they deliver bioactive substances more effectively than traditional cast films do (Lan et al. 2022).

Electrospraying functions as an electrospinning‐related method that produces droplets with nano and micro dimensions from polymer solutions through the application of high electric fields (Choukaife et al. 2025). The charged droplets solidify during flight because the solvent evaporates at high speed, which leads to the creation of nanoparticles that can either form a film layer or be incorporated into edible films (Dhiman et al. 2022). The method allows accurate control over three aspects of material properties, which include particle size and shape and the efficiency of their protective coating. The method enables materials to release antimicrobial, antioxidant, and other active substances in two different ways, which include continuous release and release triggered by external factors (Wang, Kong, et al. 2022). Electrospraying proves to be the optimal method for creating functionalized film surfaces, which enable the production of coatings containing high levels of bioactive materials (Levana et al. 2022).

Emerging techniques, which include layer‐by‐layer assembly, use controlled deposition of oppositely charged particles and nanomaterials to create multilayered films that have customized barrier and mechanical characteristics (Cai et al. 2024). The coacervation technique takes advantage of the phase separation phenomena to create polymer‐nanomaterial complex compounds, which encapsulate bioactive compounds before film formulation (Alrosan et al. 2025). The techniques enable researchers to create films which have specific release patterns while maintaining improved stability and multiple functional capabilities, but they need researchers to manage three essential elements, which include polymer behavior, solution properties, and processing conditions (Hassen and Ben Salah 2026).

## 5 Controlled Release Mechanisms in Nano‐Enabled Systems

### 5.1 Encapsulation Strategies

Electrospraying has become a versatile and efficient technique for encapsulating bioactive compounds in food and nutraceutical applications (Wang, Kong, et al. 2022). Encapsulation enables bioactive compounds, flavors, and nutrients to be incorporated into food matrices in a controlled and stable manner, thereby supporting the development of high‐quality functional foods (Biswas et al. 2022). For instance, microencapsulation has been successfully applied to incorporate omega‐3 fatty acids into baked products without affecting taste or texture (Zabot et al. 2022). Encapsulation technologies play a vital role in advancing food bio‐manufacturing by addressing key challenges related to the stability, delivery, and bioavailability of bioactive compounds (Dhiman et al. 2022). They protect sensitive ingredients such as vitamins, antioxidants, and probiotics from environmental stressors, including oxidation, moisture, and heat, ensuring their stability during processing, storage, and consumption (Feng et al. 2025). Similarly, Pickering emulsions have been used to formulate reduced‐fat mayonnaise and salad dressings that retain the sensory qualities of full‐fat products (Younis et al. 2026). In addition, encapsulation techniques enable the controlled and sustained release of functional ingredients, thereby enhancing their effectiveness and bioavailability (Multisona et al. 2025). With the growing demand for functional foods, nutraceuticals, and health‐promoting ingredients, these advanced encapsulation technologies have become central to modern innovations in food manufacturing (Wang, Kong, et al. 2022).

The polymer matrix, which consists of food‐grade biopolymers like chitosan, gelatin, zein, alginate, and starch derivatives, enables engineers to create materials that exhibit specific barrier functions, mechanical strength, and biocompatibility, so that materials inside the protective shield will be released according to planned time and location (Younis et al. 2026). Stimuli‐responsive or controlled release platforms are also provided by electrospun nanocarriers (Shi et al. 2024). Environmental factors, which include pH, moisture, temperature, and enzymatic activity, enable the controlled release of encapsulated bioactives through the modulation of polymer composition, crosslinking density, and particle morphology (Dhiman et al. 2022). The ability to continuously deliver antimicrobials and antioxidants throughout time becomes essential for food applications because it enables products to maintain their freshness and quality while extending their usable period and increasing their nutritional content (Liu, Zhu, et al. 2025). Electrospraying enables high encapsulation efficiency with even particle size distribution, which serves as a fundamental requirement for achieving reproducible results with stable performance in industrial applications (Dhiman et al. 2022).

Electrospraying offers many opportunities for additional functionality and versatility in deposition and integration with food matrix (Liu, Zhu, et al. 2025). The electrosprayed particles can be directly applied onto edible films, which can be used to create coatings and be used to distribute active compounds into drinks and solid food products (Xie, Ma, et al. 2024; Xie, Liu, and Zhang 2024). The high surface‐area‐to‐volume ratio of nanoparticles produced by electrospraying increases their solubility, bioavailability, and ability to interact with food materials, which results in better effectiveness (Nisar and Wan 2025). Electrospraying functions as an effective encapsulation method that protects bioactive compounds while enabling their scheduled release, thus serving as a valuable method for creating advanced operational food items that contain nanotechnology elements (Hao et al. 2025).

### 5.2 Release Kinetics

The release kinetics of bioactive compounds from nano‐enabled edible films determines their effectiveness in food preservation and shelf‐life extension and functional performance (Dube 2025). Release kinetics describes the rate and mechanism by which encapsulated compounds, which include antimicrobials, antioxidants, vitamins, and nutraceuticals, are released from the polymeric matrix into the food environment (Siddiqui et al. 2023). The release behavior of a film material must be thoroughly understood and controlled so it can deliver bioactive agents in a sustained and controlled manner, which also responds to external stimuli until the targeted functional results are achieved (Zhang, Xue, et al. 2025; Zhang, Qin, et al. 2025; Zhang, Zhang, et al. 2025; Zhang, Liu, et al. 2025).

The release of bioactives from edible films occurs through multiple mechanisms, which include diffusion, polymer degradation, swelling, and stimuli‐responsive triggers (Zhang, Xue, et al. 2025, Zhang, Qin, et al. 2025, Zhang, Zhang, et al. 2025, Zhang, Liu, et al. 2025). Diffusion‐controlled release occurs when the bioactive agent, while migrating away from the polymer matrix, experiences a concentration gradient during the movement (Rahman et al. 2025). Films made from hydrophilic polymers like starch, alginate, and chitosan demonstrate this mechanism because bioactive molecules move through the polymer network with time (Moeini et al. 2022). The rate of diffusion depends on four factors, which include polymer porosity, crosslinking density, molecular weight, and particle size of encapsulated nanocarriers (Kesharwani et al. 2024).

The polymer matrix of degradation‐controlled release systems disintegrates gradually through the processes of hydrolysis, enzymatic activity, and environmental factors, which lead to the controlled release of its contained materials (Herdiana et al. 2022). The method relies on biodegradable polymers, which include zein, gelatin, and poly (lactic acid), to create predictable release patterns that match the matrix degradation rate (Yi et al. 2024). Discharge due to swelling in the polymeric system is another phenomenon through which bioactive compounds may diffuse through the network when the polymer is swollen due to absorption of water from food or surroundings (Zhang, Xue, et al. 2025, Zhang, Qin, et al. 2025, Zhang, Zhang, et al. 2025, Zhang, Liu, et al. 2025). Hydrophilic matrices demonstrate high moisture sensitivity, which enables the development of moisture‐based release systems used in fresh produce and dairy products and high‐moisture food applications.

The advanced mechanism of stimuli‐responsive release utilizes environmental triggers such as pH, temperature, ionic strength, and enzymatic activity to regulate the timing and amount of bioactive compound release from edible films (Zhang, Xue, et al. 2025, Zhang, Qin, et al. 2025, Zhang, Zhang, et al. 2025, Zhang, Liu, et al. 2025). For example, pH‐responsive chitosan‐based edible films loaded with essential oils have shown enhanced antimicrobial release under acidic conditions commonly associated with microbial spoilage in meat and seafood products (Hou et al. 2023). Temperature‐responsive films containing nanoencapsulated antioxidants have been applied in fresh produce packaging, where increased temperatures accelerate the release of active compounds to reduce oxidative deterioration (Hou et al. 2023). Similarly, enzyme‐responsive edible coatings incorporating polysaccharides and protein matrices have demonstrated controlled release of antimicrobial agents in response to microbial enzyme activity during food spoilage (Dong et al. 2024). In dairy and bakery products, ionic strength‐sensitive films have also been explored to modulate the release behavior of preservatives and flavor compounds under varying salt concentrations (Kumar et al. 2026). These smart edible film systems improve preservation efficiency, extend shelf life, and reduce unnecessary release of active compounds during storage.

Scientists use mathematical models to study release kinetics because this method allows them to measure release profiles and forecast how nano‐enabled films will behave under different testing environments (Malekjani et al. 2024). The diffusion mechanisms, polymer relaxation, and dual release mechanisms are explained through the models of Higuchi and Korsmeyer‐Peppas, zero‐order and first‐order kinetics (Zhao et al. 2023). The models enable researchers to optimize polymer composition with nanocarrier loading and film thickness until they achieve their target release profile, which includes sustained release, burst release, and extended storage control release (Siddiqui et al. 2023).

### 5.3 Advantages for Food Preservation

The controlled release system from nano‐enabled edible films provides food preservation benefits that exceed what traditional packaging and additive methods can deliver (Wang, Guo, et al. 2025). The major advantages include the potential reduction of the volume of preservatives that consumer food products use (Novais et al. 2022). The bioactive compounds are released from their polymeric nanocarriers because their antimicrobials, antioxidants, and nutraceuticals are enclosed within these carriers. The carriers respond to environmental conditions such as changes in pH, moisture levels, and microbial activity (Zabot et al. 2022). The method maintains active compound concentrations at the food surface and within the matrix, enabling effective delivery without the need for high initial doses (Liu et al. 2024). In turn, the preservation of foodstuffs is tended to with increased attention to health and safety in the face of high specters of chemical preservatives, health hazards, and other regulatory concerns (Teshome et al. 2022).

Another major advantage of controlled release technology is its ability to significantly reduce the sensory impact on the final food product (Westlake et al. 2022). High concentrations of bioactive compounds such as essential oils and polyphenols can produce strong taste, aroma, and visual effects that may reduce consumer acceptance of products (Nieto et al. 2023). The nano‐enabled edible films provide encapsulation, which delivers compounds at specific times and locations while blocking direct food contact until the delivery is necessary (Meng et al. 2024). It retains the organoleptic properties of food like taste, aroma, and texture as well as features like protection against spoilage and oxidation (Sharma et al. 2024).

Controlled release also allows for antimicrobial and antioxidant activity to be maintained over the long term during storage and transport (Hou et al. 2023). Conventional preservative methods create an initial active agent concentration that exceeds the required level for their intended use but result in decreased protection during storage because active agents break down or spread out over time (Muthuvelu et al. 2023). The nano‐enabled edible films with their controlled‐release abilities sustain bioactive levels which result in effective protection against microbial growth, lipid oxidation, and enzymatic degradation (Dube 2025). This feature is particularly important for highly perishable foods such as fresh fruits and vegetables, dairy products, meat, and fish, where the continuous release of active compounds can significantly extend shelf life and reduce food waste (Meena et al. 2025).

The development of controlled‐release systems to respond to environmental triggers allows for the automatic delivery of bioactive substances when temperature, moisture conditions, and microbial growth patterns change (Zhang, Xue, et al. 2025; Zhang, Qin, et al. 2025; Zhang, Zhang, et al. 2025; Zhang, Liu, et al. 2025). The smart behavior of this system delivers two benefits by improving preservative efficiency while the system protects product safety and quality through its delivery of protective compounds at their most crucial times (Hou et al. 2023). The controlled‐release edible films help meet current consumer demand for clean‐label foods that contain only natural ingredients, which have undergone minimal processing because their total preservative load is reduced (Davidescu et al. 2025).

## 6 Antimicrobial Function of Nano‐Enabled Edible Films

### 6.1 Natural Antimicrobial Agents

The incorporation of non‐conventional food ingredients and biomolecules into edible films has attracted considerable attention due to their ability to enhance the nutritional and protective properties of food packaging systems (Castro‐Muñoz 2025). Materials such as algae extracts, insect proteins, agricultural by‐products, fruit and vegetable peels, seed residues, and microbial polysaccharides are increasingly used as sustainable alternatives in edible film production (Martins et al. 2024). These resources are rich in proteins, polysaccharides, dietary fibers, polyphenols, vitamins, and natural antioxidants that improve film functionality, nutritional value, and food preservation. Additionally, many of these biomolecules exhibit antimicrobial and antioxidant activities, helping to extend shelf life and improve food safety (Fadiji et al. 2023). Their utilization also promotes waste valorization and environmental sustainability by converting agro‐industrial residues into value‐added packaging materials. Therefore, non‐conventional biomolecules offer promising opportunities for developing multifunctional edible films with enhanced nutritional, protective, and eco‐friendly properties (Fadiji et al. 2023).

Natural antimicrobial agents have gained significant attention for their use in nano‐enabled edible films because they can kill foodborne pathogens and satisfy customer requirements for clean‐label and minimally processed products (Elbehiry and Alajaji 2026). These bioactive compounds are obtained from the plant, microbial, or animal domain. Such compounds involve essential oils, plant extracts, bacteriocins, and organic acids (Deshmukh and Gaikwad 2024). Their incorporation into biopolymer matrices or nanocarriers improves antimicrobial activity, enhances product stability, and enables controlled release, thereby extending shelf life and improving the safety of perishable foods (Hou et al. 2023).

Essential oils such as thymol, carvacrol, eugenol, and cinnamaldehyde exhibit strong antimicrobial activity against a wide range of pathogens (Khwaza and Aderibigbe 2025). These compounds exhibit antibacterial, antifungal, and anti‐yeast activity by disrupting microbial cell membranes, inhibiting enzyme activity, and interfering with intracellular functions (Alhantoobi et al. 2025). The antimicrobial and antioxidant activities of plant extracts, which include green tea polyphenols, grape seed extract, and rosemary extract, demonstrate powerful effects (Salih et al. 2024). The chemicals are high in phenolics that act together with flavonoids to prevent microbial spoilage and oxidative deterioration (Table 2).

**TABLE 2:** Common natural antimicrobial agents used in edible films, their sources, and their primary mechanisms of action.

Natural antimicrobial agent	Source	Target microorganisms	Mechanism of action	References
Thymol, Carvacrol, Eugenol	Essential oils (thyme, clove, oregano)	Bacteria, fungi, yeasts	Disrupts cell membranes and inhibits enzymes	de Almeida et al. (2023)
Cinnamaldehyde	Cinnamon bark oil	Bacteria, fungi	Inhibits cell wall synthesis, protein denaturation	Nirmal et al. (2023)
Green tea polyphenols	*Camellia sinensis* leaves	Bacteria, fungi	Oxidative stress induction, enzyme inhibition	Maslov et al. (2022)
Grape seed extract	*Vitis vinifera* seeds	Bacteria, fungi	Membrane disruption, antioxidant activity	Krasteva et al. (2023)
Nisin	*Lactococcus lactis* (bacteriocin)	Gram‐positive bacteria	Pore formation in the cell membrane	Thanjavur et al. (2022)
Pediocin	*Pediococcus* spp.	Gram‐positive bacteria	Disrupts cell wall integrity, inhibits growth	Fugaban et al. (2022)
Lactic acid	Fermentation/metabolite	Bacteria, fungi	Lowers pH, disrupts metabolic processes	Liu et al. (2025)
Citric acid	Citrus fruits	Bacteria, fungi	Acidifies the environment, chelates metal ions	Al‐Hajani et al. (2022)

Certain lactic acid bacteria produce peptide‐based antimicrobial agents such as nisin and pediocin that are referred to as Bacteriocins (Singh et al. 2024). The preservation method shows its highest effectiveness when applied to Gram‐positive bacteria. The method finds its most effective application in preserving dairy products, meat, and seafood items (Todorov et al. 2022). Organic acids like lactic, citric, and acetic acids lower the pH of the food environment, creating unfavorable conditions for microbial proliferation (Sorathiya et al. 2025). The agents in nano‐enabled edible films offer three key advantages: controlled release, improved solubility, and enhanced stability, ensuring prolonged antimicrobial activity.

The use of natural antimicrobial compounds in your food product's edible film system will extend its shelf life because the film possesses water solubility, low evaporation rates, and controlled‐release functions (Fadiji et al. 2023). The system enables consumers to obtain products that retain their natural state through minimal processing and natural preservation methods, without the use of chemical preservatives (Chakraborty and Dutta 2022). The films serve multiple food applications because they can be used with various food products, which include fruits and vegetables, meats and dairy products, and baked goods. These films have wide food applications and can be used with various products, including fruits, vegetables, meat, dairy products, and baked goods (Lohita and Srijaya 2024).

### 6.2 Nanoparticle‐Based Antimicrobial Mechanisms

As shown in Table 3, each nanomaterial targets a specific microorganism, making them highly useful in food preservation applications (Biswas et al. 2022). Nanomaterials are capable of enhancing the long‐term antimicrobial activity of edible films, acting as well in maintaining the integrity of bioactive compounds (Hassanzadeh et al. 2025). Alternatively, bioactive agents could securely be released from edible films with controlled release timers using a set amount of nanomaterials or nanocomposites (Hashemi et al. 2023). The above effect explains why nanomaterials are proving to be a viable solution, both for the sustenance of quality and prolongation of shelf‐life (Shouket et al. 2023). Nanomaterials show their antiseptic and antimicrobial properties through multiple mechanisms, which include disrupting microbial cell membranes, creating free radicals, and interfering with cellular metabolism, and inhibiting nucleic acids and proteins (Nie et al. 2025).

**TABLE 3:** Nanoparticle types used in edible films, their effective doses, target microorganisms, applicable food types, and mechanisms of action.

Nanoparticle type	Dose	Target microorganisms	Type of food	Mechanism of action	References
Silver nanoparticles (AgNPs)	50–200 ppm	*E. coli* , *L. monocytogenes* , *S. aureus* , Candida spp.	Meat, poultry, dairy	Cell membrane disruption, ROS generation, DNA/protein damage	Michalak et al. (2022)
Zinc oxide nanoparticles (ZnO NPs)	0.5%–2% w/w	Salmonella spp., *E. coli* , Aspergillus spp.	Fruits, vegetables, and bakery	ROS‐mediated oxidative stress, membrane damage	Gökmen et al. (2024)
Copper oxide nanoparticles (CuO NPs)	0.5%–1% w/w	*S. aureus* , *E. coli* , Penicillium spp.	Dairy, meat		Gholizadeh et al. (2025)
Titanium dioxide nanoparticles (TiO2 NPs)	0.1%–0.5% w/w	Bacteria, fungi	Fruit coatings, beverages	Photo‐catalytic ROS generation, oxidative damage	Bastardo‐Fernández et al. (2024)
Chitosan nanoparticles	1%–3% w/w	*E. coli* , *L. monocytogenes* , molds	Fresh produce, seafood	Electrostatic interaction with the cell wall, membrane permeabilization	Karimzadeh et al. (2023)

A large number of research works have emphasized various kinds of metallic nanoparticles (NPs) with their antimicrobial activities (Skłodowski et al. 2023). These types of NPs include silver (Ag), zinc oxide (ZnO), and copper oxide (CuO) NPs (Mathimaran et al. 2024). Ag NPs establish two separate bactericidal mechanisms through their ability to damage microbial cell membranes while creating reactive oxygen species (ROS), which interrupt the replication process of nucleic acids and the synthesis process of proteins (Do et al. 2025). ZnO NPs generate reactive oxygen species, which create active surfaces that produce oxidative stress to destroy microorganisms by their membrane destruction and cellular damage via their interaction with microbial surfaces (Yan et al. 2023). Copper oxide NPs perform as potent inhibitors of growth for microbes extensively through oxidative actions combined with compounds that act as inhibitors of enzymes (Shehabeldine et al. 2023). Biopolymer‐stabilized nanoparticles function as nanoemulsions that enable the encapsulation of antimicrobial agents and their subsequent controlled release, thereby maximizing the effectiveness of the antimicrobial agents while preserving food quality (Joolaei Ahranjani and Ferrentino 2025).

The process of embedding nanoparticles inside edible films and packaging materials enables the sustained delivery of active ingredients, which include natural preservatives that require high concentrations for effective use, while simultaneously improving food product quality and safety (Kumar et al. 2022). The small size of the nanoparticles provides greater contact with the microorganism, which improves the nanomaterials' capacity to eliminate both pathogens and spoilage organisms (Jadhav et al. 2023). The combination of nanoparticles with natural antimicrobial agents produces a compound effect that extends their antimicrobial power while decreasing the chance of microorganism resistance development (Ribeiro et al. 2022). Using edible films prepared with nanoparticles thus shows progression towards active food packaging as an innovative concept with much enhanced antimicrobial capability in diverse food types (Kumar et al. 2025).

### 6.3 Synergistic Effects

The research shows that nano‐enabled food packaging combines electrosprayed nanocarriers with edible films, demonstrates better antimicrobial properties and food preservation capabilities through the specific characteristics of electrospray technology (Younis et al. 2025). The system produces nano‐sized particles or microcapsules, which possess an exceptionally high surface area volume ratio; the nano‐enabled food packaging materials demonstrate their strength and barrier properties and controlled delivery characteristics (Muthu et al. 2025). Perishable foods experience extended shelf‐life because electrospray technology, together with nano‐enabled food packaging methods, creates better protection against foodborne pathogens and spoilage microorganisms (Pandhi et al. 2023).

Electrospraying serves as a specialized technique that enables the exact process of bioactive compound containment through the creation of polymer carriers that contain natural antimicrobials, essential oils, plant extracts, synthetic preservatives, and metallic nanoparticles (Shavronskaya et al. 2023). The electrospraying process produces particles that have a uniform particle size distribution while their shapes stay under scientific control to create stable particles that protect labile compounds from thermal damage, oxidative damage, and photodegradation (Feng et al. 2023). The electrosprayed nanoparticles deliver two benefits because they create a constant distribution of active compounds in edible films while their antimicrobial properties extend uniformly throughout all sections of the film (Levana et al. 2022).

Multiple mechanisms work together to create a combined antimicrobial effect. The antimicrobial treatment of nanoparticles, silver, zinc oxide, and chitosan demonstrates its antimicrobial properties through three mechanisms, which include membrane disruption in bacteria, the production of reactive oxygen species, and the identification of metabolic and genetic pathways (Singh et al. 2025). The antimicrobial agents contained in bioactive nano‐carriers developed through electrospray processing have two release methods, which include controlled release and on‐demand release. The sustained activity of these agents can be maintained through their extended storage period (Petrovic and Barbinta‐Patrascu 2023). The two antimicrobial systems working together create enhanced antimicrobial protection, enabling the use of smaller preservative amounts that produce less sensory effects on the final product and meet consumer demand for clean label products (Bensid et al. 2022).

Research has demonstrated that edible thin films with electrosprayed nano‐sized carriers deliver significantly greater antimicrobial effectiveness than thin films containing free bioactive compounds that have not been encapsulated (Plati and Paraskevopoulou 2022). The study found that AgNPs, which researchers encapsulated through the electrospraying method and combined with chitosan thin film, showed more effective reduction of *Escherichia coli* and *Listeria monocytogenes* bacteria on fresh vegetables than standard antimicrobial units (Bizymis et al. 2023). The essential oil‐loaded nanoparticles present in nano‐enabled thin films demonstrated their ability to inhibit mold and fungal spores for an extended duration when applied to baked goods and dairy products (Yang et al. 2025). The electrosprayed nano‐sized carriers provide a very large surface‐area‐to‐volume ratio that enables bacteria to contact the active agent in each carrier at a high level (Yan et al. 2022). The format uses a polymer matrix system that produces sustained release capabilities to deliver better antimicrobial results and longer protection against microbial spoilage (Hou et al. 2023).

The combination of electrosprayed nanocarriers with nano‐enabled films allows for customized release profiles that meet specific food preservation requirements through controlled changes in polymer composition, particle size, and cross‐linking density, enabling immediate and sustained and stimulus‐responsive antimicrobial release (Younis et al. 2025). The system needs to protect its bioactive materials through intelligent systems because it requires bioactive material release to occur at specific times and locations to maintain its operational safety (Bhatlawande et al. 2024).

## 7 Applications in Food Preservation

### 7.1 Fruits and Vegetables

Edible Films with nanotechnology can improve the life cycle of fresh fruits and vegetables by retaining fresh quality (Sharma et al. 2025). Fresh produce carries naturally very high moisture; hence, fresh produce has a very limited shelf life, primarily due to active metabolism and very high levels of microbial spoilage (Palumbo et al. 2022). Traditional methods used to store fresh produce include refrigeration, modified atmospheric packaging, and synthetic coatings, which fail to provide long‐term preservation because their effectiveness lasts only for a limited period (Ju, Zhao, Zhu, and Li 2025; Ju, Zhao, and Vishnuvarthanan 2025). Edible films integrated with nanomaterials can serve as an environmentally friendly, functional, and controlled‐release model for postharvest protection (Wang, Guo, et al. 2025).

The protection provided for fresh produce can be derived from natural antimicrobials in synergy with antioxidants, nanoparticles, and biopolymer matrices (Vieira et al. 2022). Researchers created chitosan films that included silver nanoparticles and zinc oxide nanoparticles to develop antimicrobial protection against *Escherichia coli* , *Listeria monocytogenes* , and *Botrytis cinerea* on strawberries, tomatoes, and grapes (Ray et al. 2022). The bioactive compounds that exist at nanoscale dimensions enable them to interact directly with microbial cells that exist on fruit and vegetable surfaces. Therefore, nano‐dispersed bioactive compounds demonstrate superior microbial growth inhibition compared to traditional coatings (Dey et al. 2024).

The nano‐enabled films protect microorganisms through their protective layer, which also provides controlled release of antioxidants that include essential oils and plant‐based polyphenols, reduce oxidative stress while extending the shelf life of fresh fruit and vegetable products (Akbari et al. 2022). Essential oil‐loaded nanoemulsions, which researchers incorporated into alginate and gelatin films, maintained their original color and texture and vitamin C content during storage in refrigerated conditions (Wang, Xue, et al. 2024). The nanoparticles' extremely large surface area compared to their total volume enables bioactive molecules to diffuse through their structure for extended periods, extending their capacity to function as both antimicrobial agents and antioxidants (Heidari et al. 2022). Essentially, these manufactured products offer an effective means of producing high‐quality, perishable, edible products with extended shelf life.

The active protection system, together with nano‐enabled films that enhance coating barrier capabilities, leads to decreased water loss, gas exchange, and respiration rates of the system (Wang, Guo, et al. 2025). The combination of polymers, starch, alginate, pectin, and cellulose derivatives with nanoparticles produces semi‐permeable films which control moisture loss to protect leafy vegetables and fruits from wilting and desiccation (Tayel et al. 2024). The functional barrier keeps products fresh while it protects against mechanical damage and prevents products from shrinking during transportation and display in retail stores (Turan et al. 2024).

The creation of nano‐enabled edible films currently allows for their development to achieve responsiveness towards environmental conditions including pH levels, temperature changes, and microbial growth patterns (Muthu et al. 2025). pH‐sensitive films use their ability to detect pH changes for their antimicrobial release mechanism because they need organic acid levels to rise from microbial activities before showing their protective effects against spoilage (Hou et al. 2023). Climacteric fruits, which include bananas, mangoes, and tomatoes, benefit from this adaptive behavior because their postharvest ripening process requires protection against their high vulnerability to microbial contamination (Chan et al. 2025).

### 7.2 Meat and Poultry Products

The use of nano‐enabled edible films has proven to be an effective method for meat and poultry product preservation because they reduce lipid oxidation and microbial spoilage and discoloration leads to improved safety and sensory quality (Table 4). The high moisture content and rich nutrient composition of fresh and processed meat make them highly perishable because they can easily undergo oxidative reactions and microbial contamination (Mediani et al. 2022). Traditional preservation methods, which include vacuum packaging and refrigeration, provide only limited protection, while synthetic additives create potential health risks (Teshome et al. 2022). Nano‐enabled films provide an active and controlled‐release system that enhances product stability while minimizing the use of chemical preservatives (Dube 2025).

**TABLE 4:** Nano‐enabled edible films applied to meat and poultry products.

Nanoparticle/Bioactive	Dose/Concentration	Target microorganisms	Type of meat	Mechanism of action	References
Silver nanoparticles (AgNPs)	50–150 ppm	*E. coli* , *L. monocytogenes*	Chicken, beef	Membrane disruption, ROS generation, DNA/protein damage	Hassanen et al. (2023)
Zinc oxide nanoparticles (ZnO NPs)	0.5%–1.5% w/w	Salmonella spp., spoilage bacteria	Pork, poultry	Oxidative stress, membrane disruption	Sasidharan et al. (2024)
Chitosan nanoparticles	1%–3% w/w	*E. coli* , *S. aureus* , molds	Beef, lamb	Electrostatic interaction, membrane permeabilization	Ghadami and Esmaeilpour (2025)
Essential oil‐loaded nanoemulsions	0.5%–2% w/w	*E. coli* , Salmonella, Listeria	Minced meat, sausages	Disruption of cell membranes, sustained release of bioactive agents	Hashemi et al. (2024)
Nanoemulsion of antioxidants	0.5%–1% w/w	Lipid oxidation	Processed meat	Controlled release of antioxidants, delayed rancidity	Snoussi et al. (2022)

The use of metallic nanoparticles (AgNPs and ZnO NPs) and natural antimicrobials (essential oils and plant extracts) together with antioxidants in edible films results in enhanced protection against microbial growth and better preservation of meat and poultry food products (Vieira et al. 2022). The controlled release of antioxidants polymeric matrices contain acts as a solution to reduce the rate of lipid oxidation, which serves as the primary source for producing rancidity and color degradation (Musakhanian et al. 2022). Nanoparticles improve antimicrobial effectiveness through their ability to increase contact between surfaces and microbial cells while they also disrupt cellular membranes and produce reactive oxygen species (ROS), which prevent the growth of *Listeria monocytogenes* , *Escherichia coli* , *Salmonella* spp., and spoilage bacteria on meat products (Mondal et al. 2024). Meat and poultry products maintain their color, texture, and flavor through the use of nano‐enabled edible films, which package their products during refrigeration and modified atmosphere storage (Dube 2025). This active packaging approach not only improves shelf stability but also reduces reliance on synthetic preservatives, meeting consumer demand for natural and minimally processed foods (Nunes et al. 2023).

### 7.3 Dairy and Bakery Products

The high moisture content, together with the sugar and fat content of dairy and bakery products, creates a situation in which microorganisms can easily degrade these products (Noshirvani and Abolghasemi Fakhri 2025). Nano‐enabled edible films protect dairy food products from microbial and oxidative damage through their mechanism of controlled antimicrobial and antioxidant agent release, thus extending product shelf life (Cheng et al. 2024).

In cheeses, yogurts, and milk products, microbial contamination may yield issues regarding flavor, texture, and safety (Dash et al. 2022). Antimicrobial nanoparticles and essential oil‐loaded nanocarriers function in edible films of dairy and bakery products to achieve controlled bioactive agent release, which specifically targets the spoilage microorganisms *Listeria monocytogenes* , *E. coli* , and *lactic acid bacteria* (Guidotti‐Takeuchi et al. 2022). For example, thin films of chitosan containing silver nanoparticles are being used to protect the surface of cheese against the growth of mold and bacterial contamination without affecting taste and texture (Mikky et al. 2024). Antioxidant nanocarriers prevent the oxidation of milk fat (Du, Wang, and Zheng 2023; Du, Sun, et al. 2023).

Bakery products become vulnerable to mold growth and staling. The nano‐based films contain essential oils of clove and cinnamon and nanoparticles, function as active barriers that prevent microbial growth and control moisture movement (Nair et al. 2022). Controlled release systems ensure the gradual release of antimicrobials to prevent rapid spoilage (Hou et al. 2023). For example, eugenol and cinnamaldehyde nanoemulsions stabilized by starch and cellulose films showed effective mold protection in bread, cakes, and muffins for more than 3 days when compared to standard packaging (Mokhtari et al. 2024).

## 8 Safety, Toxicological, and Regulatory Considerations

The implementation of electrosprayed nanocarriers and nano‐enabled edible films for food preservation purposes brings significant benefits to the food industry. However, this technology introduces critical safety issues and toxicological risks that must be addressed (Mahato et al. 2024). The small size of the particles, together with their increased reactivity and bioaccumulation properties, creates potential dangers that require proper evaluation for assessment (Hund‐Rinke et al. 2022). Thus, risk assessment is one of the most prevailing factors in the commercialization of these high‐tech food applications technologies.

Biological systems respond to nanoparticles, including silver (AgNPs), zinc oxide (ZnO NPs), titanium dioxide (TiO_2_ NPs), and copper oxide (CuO NPs), in distinct ways because their nanoscale form differs from their bulk material state (Dianová et al. 2022). Due to their size, they can easily enter cells, which can cause oxidative stress, DNA damage, or inflammation (Min et al. 2023). But when these nanoparticles are mixed with polymer matrices and electrosprayed, they become immobilized or embedded (Khdary et al. 2023). The practice decreases the likelihood of people making direct contact with surfaces and can lead to systemic health hazards. Biopolymer carriers, including chitosan, alginate, and cellulose derivatives, provide nanoparticles with enhanced safety because they stop nanoparticles from entering food products (Kučuk et al. 2023).

The regulatory agencies also stress the necessity of testing this technology when it comes to evaluating the capacity of nanoparticles and bioactive compounds to migrate from films into food (Gupta et al. 2024). In the release of nanoparticles, the size matters more than anything else, but also concentration and composition of the films, storage temperature, and type of food come into play (Gao et al. 2025). Controlled‐release nanocarriers are designed to slowly release bioactive compounds, which they contain on the surface of food, to stop people from consuming excessive amounts of those compounds (Westlake et al. 2022). The edible films need to undergo migration tests, in vitro cytotoxicity tests, and in vivo experiments to demonstrate their compliance with food contact material safety requirements (Kalliampakou et al. 2025).

Internationally, regulatory agencies have developed specific guidelines for the safe application of nanomaterials in food packaging and nano‐enabled edible films (Singh and Kumar 2023). The FAD recommends comprehensive safety evaluations of nanomaterials used in food‐contact substances, including assessments of particle size, migration potential, toxicity, stability, and consumer exposure before market approval (Singh and Kumar 2023). Similarly, the European Food Safety Authority requires detailed physicochemical characterization, risk assessment, migration studies, and toxicological evaluation of engineered nanomaterials incorporated into edible films and food packaging systems (Chávez‐Hernández et al. 2024). EFSA also emphasizes the need to evaluate nanoparticle behavior during digestion and long‐term dietary exposure. In India, the Food Safety and Standards Authority of India has introduced guidelines focusing on labeling, safety assessment, permissible migration limits, and regulatory approval for nanotechnology‐based food products and packaging materials (Bumbudsanpharoke and Ko 2015). These regulations collectively aim to ensure that nano‐edible films do not release harmful levels of nanoparticles into food while maintaining product safety, functionality, and consumer health protection (Chávez‐Hernández et al. 2024).

## 9 Challenges and Limitations

The advanced technology of electrospraying and nano‐enabled edible films for active food preservation faces multiple obstacles including technical challenges, economic limitations, and regulatory requirements, together with safety concerns that must be solved before the technology can achieve full implementation (Jamwal and Mittal 2024). Addressing these constraints is crucial because the technology is feasible and secure for adoption.

The large‐scale production and scalability of the process towards commercial operation is a formidable challenge (Mahajan et al. 2025). Electrospraying allows laboratories to achieve high efficiency because the method gives researchers complete control over particle dimensions, particle form, and particle coating effectiveness (Jayaprakash et al. 2023). The electrospray process faces industrial scaling challenges because it operates with low production rates, requires excessive energy, and demands precise control across multiple process variables including voltage, flow rate, and solution characteristics (Dhiman et al. 2022). Continuous processing and multi‐nozzle systems are being developed, but scalability is still a challenge.

Cost considerations also present a significant limitation. The production expenses rise when specialized equipment and high‐purity biopolymers and engineered nanomaterials are used instead of standard food packaging materials (Almotairy et al. 2026). The process of creating commercial products for the food industry will face obstacles because the production expenses will increase when metallic nanoparticles and nano‐carriers are used (Sahoo et al. 2022). Economic feasibility studies and optimization of material usage are therefore necessary to minimize costs while ensuring functional performance (Mahajan et al. 2025). The second major challenge that needs to be addressed is consumer perception and product acceptance. Negative consumer perceptions about the safety of nanotechnology in foods could act as a barrier to market acceptance (Liao et al. 2025). The word nano is generally linked to health risks, despite the potential benefits of nano‐enabled edible films (Wang, Guo, et al. 2025).

The commercialization process becomes more difficult because of uncertain regulations. The existing food safety regulations in most areas do not have a clear set of rules specifically designed for the nanomaterials used in edible films and coatings (Ju, Zhao, Zhu, and Li 2025; Ju, Zhao, and Vishnuvarthanan 2025). Essential information about nanoparticle characterization, migration, toxicology, and exposure risks is required by the regulatory bodies relating to this nanoparticles' applications (Isibor 2024). The lack of standardized global regulations makes it difficult for companies to get their products approved and commercialized (Chávez‐Hernández et al. 2024).

The process requires extensive examination through toxicological studies and migration studies, which lead to additional research expenditures. The biopolymers used in edible films achieve general recognition as safe (GRAS) status, but scientists still need to investigate how nanomaterials interact with complex food systems and human gastrointestinal tract systems (Bizymis and Tzia 2022). The safety of consumers requires detailed research on how edible films transfer nanoparticles to food and how those nanoparticles lead to bioaccumulation and long‐term toxic effects (Akbari‐Alavijeh et al. 2022).

The research needs to assess how nano‐enabled edible films perform during actual storage and distribution operations. The environmental conditions, which include temperature changes, humidity levels, light exposure, and mechanical stress, will impact the performance of edible films through three main effects (Wang, Guo, et al. 2025). It is essential to ensure the long‐term stability of nano‐enabled edible films without compromise in their functionality (Mahato et al. 2024).

## 10 Future Horizons and Research Directions

Electrospraying together with nano‐enabled edible films has emerged as a growing research field that aims to develop packaging materials that offer intelligent, sustainable features and multiple packaging capabilities (Jamwal and Mittal 2024). Researchers will focus their future studies on upgrading material design and scalability, safety, and functional performance to meet industrial and regulatory requirements (Aguilar‐Pérez et al. 2023).

Smart and responsive food packaging is one of the most promising areas for scientific studies for the future (Said and Lee 2025). Future films could be developed to release antimicrobial or antioxidant agents because they need to respond to specific spoilage signals, which they detect through pH, temperature, enzyme, and moisture‐sensitive nanocarriers (Hou et al. 2023). On‐demand release systems, such as applications, would do just fine and require less active agent to be delivered than other ways (Du, Wang, and Zheng 2023; Du, Sun, et al. 2023). Electrospraying could also incorporate sensor technologies to provide real‐time monitoring of food quality and safety (Ramachandraiah et al. 2025).

Sustainable and biobased nanomaterials are thought to define future questions and areas of research (Saud et al. 2024). Research currently focuses on two main areas: biodegradable materials and renewable polymer materials, and plant‐based or microbial‐based green‐synthesized nanoparticles (Aswathi et al. 2023). These materials not only have lower environmental impact but also improve consumer acceptance and regulatory compliance (Chávez‐Hernández et al. 2024). Natural nanocarriers together with protein‐based nanoparticles, polysaccharide‐based nanoparticles, and food‐derived bioactive compounds will serve as fundamental components for advancing clean‐label preservation methods (Santiesteban‐López et al. 2022).

Another key research area focuses on advancing electrospraying technology to larger operational scales. Future research should focus on developing high‐throughput energy‐saving electrospraying technology through the creation of multi‐nozzle and needleless electrospraying systems, which support continuous operation (Bhushani and Anandharamakrishnan 2014). For commercialization, one will have to blend electrospraying techniques with conventional coating, extrusion, or packaging‐based activities (Dhiman et al. 2022).

The research should examine complete toxicological studies together with migration tests and long‐term exposure tests to determine safety. Scientists need advanced testing systems to include in vitro research methods, in vivo experimentation, and complete life cycle studies to investigate how nanoparticles interact with food and human bodies (Cao et al. 2016). Standardized tests and synchronized international regulation will be required for the safe commercialization of nano‐printed edible films (Muthu et al. 2025).

From this viewpoint, the application range of edible nano‐enabled films broadens and is seen as an important matter for future research (Mahato et al. 2024). These films have the ability to create personalized nutrition solutions, also enable precise delivery of active substances, and decrease food waste throughout supply chains (Xie, Ma, et al. 2024; Xie, Liu, and Zhang 2024). Future research collaboration between food scientists, material scientists, toxicologists, and regulators will be necessary to translate laboratory‐scale innovations into practical food system applications.

## 11 Conclusion

The integration of nanotechnology into edible film systems has opened new possibilities for improving food preservation and safety. Nano‐enabled edible films, developed from natural biopolymers and functional nanomaterials, demonstrate enhanced barrier properties, mechanical performance, and bioactive delivery compared to conventional edible coatings. These improvements address key limitations associated with traditional preservation approaches, including rapid quality deterioration and excessive use of chemical additives. Electrospraying has emerged as an effective technique for the fabrication and functionalization of nano‐enabled edible films, particularly due to its ability to encapsulate sensitive bioactive compounds under mild conditions and to control their release behavior. When applied to food systems, electrosprayed nano‐carriers provide targeted antimicrobial and antioxidant protection without adversely affecting sensory quality. Despite their promising performance, further research is required to overcome challenges related to scale‐up, regulatory compliance, and safety assurance. Establishing standardized evaluation methods and clear regulatory guidelines will be critical for industrial adoption. Overall, the synergistic application of electrospraying and nano‐enabled edible films offers a viable and innovative approach for developing sustainable and intelligent food preservation systems.

## Author Contributions

Messenbet Geremew guided the study and oversaw data collection. Messenbet Geremew, Eshetie Gelagay, and Desye Alemu provided technical comments, revised the manuscript, and approved the final version for publication. Each author's contributions were essential to the success and integrity of the research study.

## Funding

The authors have nothing to report.

## Conflicts of Interest

The authors declare no conflicts of interest.

## Data Availability

The data that support this review are available from the corresponding author upon reasonable request.
